# Analysis of heart rate as a predictor of changes in heart rate variability in children

**DOI:** 10.1590/1984-0462/2025/43/2024045

**Published:** 2024-10-28

**Authors:** Paulo César Trindade Costa, Adélia da Costa Pereira de Arruda, Vinícius José Baccin Martins, José Luiz de Brito Alves

**Affiliations:** a Universidade Federal da Paraíba, João Pessoa, PB, Brazil.

**Keywords:** Heart rate, Autonomic nervous system, Obesity, Children, Prediction, Frequência cardíaca, Sistema nervoso autônomo, Obesidade, Crianças, Predição

## Abstract

**Objective:**

To evaluate the predictive validity and cut-off point of heart rate and blood pressure on heart rate variability (HRV) changes in children with and without obesity.

**Methods:**

This study included 125 children, of whom 41 were normal weight and 84 were obese. Anthropometry, blood pressure, heart rate, and HRV were measured using an electronic scale and stadiometer, a sphygmomanometer, and HRV monitor. In addition, the receiver operating characteristic (ROC) curve was obtained by statistical analysis of the data.

**Results:**

Heart rate proved to be a good predictor for changes in the square root of the mean of the square of the differences between consecutive NN intervals (RMSSD) parameter in students of both sexes for the normal-weight group (ROC 0.89; 95%CI 0.77–1.00) and obesity (ROC 0.90; 95%CI 0.83–0.97). In addition, the heart rate cut-off point for alterations in the RMSSD parameter for normal-weight boys was 93 bpm (sensitivity 100.00% and specificity 87.50%) and for boys with obesity, the established cut-off point was 91 bpm (sensitivity 94.74% and specificity 63.64%). Heart rate also proved to be a good predictor considering low-frequency/high-frequency ratio (LF/HF) and standard deviation of long-term continuous NN intervals /standard deviation of the instantaneous variability of continuous NN intervals in the Poincaré graph ratio (SD2/SD1). Systolic and diastolic blood pressures were good predictors in more specific stratifications and, therefore, can be used in some cases.

**Conclusions:**

The predictive validity of heart rate was shown to be at a good level, with high sensitivity and acceptable specificity for the cut-off points according to the different analyses stratified by gender and nutritional status. In this sense, health professionals will be able to use heart rate to estimate cardiovascular risk in children of different sexes and nutritional status.

## RESUMO

**Objetivo:**

Avaliar a validade preditiva e o ponto de corte da frequência cardíaca e da pressão arterial nas alterações da variabilidade da frequência cardíaca (VFC) em crianças com e sem obesidade.

**Métodos:**

Foram incluídas 125 crianças neste estudo, sendo 41 com peso normal e 84 com obesidade. Antropometria, pressão arterial, frequência cardíaca e VFC foram medidas por meio de balança eletrônica e estadiômetro, esfigmomanômetro e monitor de VFC. Além disso, a curva característica de operação do receptor (ROC) foi obtida pela análise estatística dos dados.

**Resultados:**

A frequência cardíaca mostrou-se um bom preditor de alterações no parâmetro de raiz quadrada da média do quadrado das diferenças entre os intervalos NN consecutivos (RMSSD) em escolares de ambos os sexos para o grupo de peso normal (ROC 0,89; IC95% 0,77–1,00) e obesidade (ROC 0,90; IC95% 0,83–0,97). Além disso, o ponto de corte da frequência cardíaca para alterações no parâmetro RMSSD para meninos com peso normal foi de 93 bpm (sensibilidade 100,00% e especificidade 87,50%), e para meninos com obesidade, o ponto de corte estabelecido foi de 91 bpm (sensibilidade 94,74% e especificidade 63,64%). A frequência cardíaca também se mostrou um bom preditor considerando os índices da relação baixa frequência/alta frequência (LF/HF) e razão desvio padrão de intervalos NN contínuos de longo prazo/desvio padrão da variabilidade instantânea de intervalos NN contínuos na relação gráfica de Poincaré (SD2/SD1). As pressões arteriais sistólicas e diastólicas foram bons preditores em estratificações mais específicas e, portanto, podem ser utilizadas em alguns casos.

**Conclusões:**

A validade preditiva da frequência cardíaca mostrou-se em bom nível, com alta sensibilidade e especificidade aceitável para os pontos de corte, conforme as diferentes análises estratificadas por sexo e estado nutricional. Desta forma, os profissionais de saúde poderão utilizar a frequência cardíaca para estimar o risco cardiovascular em crianças de diferentes sexos e estado nutricional.

## INTRODUCTION

Childhood obesity currently affects 39 million children worldwide and its prevalence has increased at an alarming rate, with the highest incidence observed in economically disadvantaged countries such as Brazil.^1, 2^ Obesity is considered a risk factor for the development of cardiovascular and metabolic diseases and, once established in childhood, tends to persist into adulthood.^3, 4, 5, 6^

Children with overweight or obesity exhibit an increased risk of cardiovascular disorders early in life, as evidenced by sympathetic overactivity, vagal withdrawal, increased blood pressure (BP), and resting heart rate (HR).^7,8,9^ Heart Rate Variability (HRV) is a non-invasive method that quantifies the variation between consecutive heartbeats and is a commonly used and validated measure to assess cardiac autonomic function using time, frequency, and non-linear domain indices to quantify parasympathetic and sympathetic modulation.^10^

Increased HRV represents efficient autonomic activity. On the other hand, decreased HRV generally represents an abnormal and inadequate adaptation of the autonomic nervous system, which may indicate the presence of autonomic dysfunction.^11, 12^ In children, reduced HRV has been associated with obesity^13, 14^ and its complications^15^ proving to be a risk factor for cardiometabolic diseases and potentially early mortality.^13^ Therefore, regular monitoring of the HRV measurements may allow the identification of early physiological warning signs of cardiovascular disease.

Electrocardiogram Holter monitor, or chest strap is commonly used to check HRV.^16,17,18^ However, its use is not very accessible and has been mainly limited to scientific research. Furthermore, its application is not practical and requires data processing and analysis by specialists. In this sense, the use of HR or BP measurements can offer a convenient and low-cost screening method for regular monitoring of the autonomic nervous system health in children. However, the predictive validity of HR or BP for HRV measures has not been previously evaluated. Therefore, the aim of the present study was to evaluate the predictive validity and cut-off point of HR and BP in HRV changes in children with and without obesity. Using the predictive value or cut-off point for HR or BP, health professionals will be able to identify the potential risk of autonomic dysfunction in children.

## METHOD

This cross-sectional study was based on data from the project “Physical training and nutrition education as strategies to modulate the gut microbiota and metabolic, inflammatory, and cardiovascular parameters in schoolchildren with obesity: a cooperation for a multicenter study” (NUTRO Study). The main objective of NUTRO is to evaluate the effectiveness of a multicomponent intervention on cardiometabolic parameters in children with obesity.

The sample size was calculated using the following parameters: size of the reference population equal to 23,861 children from the 1^st^ to the 5^th^ year of Fundamental I (elementary school), prevalence of the obesity outcome equal to 13.30%,^19^ 95% confidence interval (CI), test power of 80.00%, and design effect (deff) equal to 1. Based on these parameters, the minimum size of the original sample was set at 76 children. Potential losses were compensated by increasing the sample size by 20.00%.

The sample was decided for convenience. Ten schools were selected from the four geographical regions of the community (North, South, East, and West). A nutritional screening of all children enrolled in the school was conducted to determine children for the study. To diagnose the nutritional status, weight and height measurements were taken and then the students were classified according to the World Health Organization (WHO) cut-off points.^20^

The study included normal-weight and obese children, aged 7–11 years, of both sexes, and who were duly enrolled in the municipal education network of João Pessoa, Paraíba (PB), Brazil. However, children who had any motor or psychological disorders that made it impossible for them to perform the tests were excluded.

The body weight was measured with an electronic scale (Omron^®^ HBF-514C) and height was assessed using a stadiometer (alturaexata^®^). Nutritional status, using body mass index for age (BMI/A) and sex according to WHO standards, was determined through Anthro Plus (version 1.0.4; WHO). Children were classified into normal weight (≥ -2 Z score ≤ +1); overweight (> +1 Z score ≤ +2); and obesity (Z score > +2).^20^

BP and HR were measured with a digital sphygmomanometer (Omron healthcare^®^ HBP-1100) validated for use with children according to the Brazilian Society of Cardiology. After an initial stabilization period (5 minutes), three consecutive measurements were taken for each child at 1-minute intervals, and the average values were used in the analysis.

All tests were conducted with the participant at rest and in the supine position. The recordings were performed in the morning in a quiet room. Short-term recording was completed in ten minutes with the children in a quiet state and breathing normally at tidal volume. Through wireless transmission, a smartphone (IPhone12) was used to record the RR intervals on an application (Elite HRV LLC, Asheville, NC, USA, Release 4.0.2, 2018). An elastic belt of adjustable size was placed comfortably around the participant’s chest and a transmitter (Polar model H10, Polar Electro, Finland) was attached to the front at the level of the xiphoid process. Previous studies have shown that the smartphone app and chest strap (Polar) provide excellent electrocardiogram compliance for all variables in the time domain, frequency domain, and nonlinear indices.^18, 21^

The predictive power of HR for changes in HRV was assessed using receiver operating characteristic (ROC) curves. First, the total area under the ROC curve between HR, systolic blood pressure (SBP), and diastolic blood pressure (DBP), and markers of changes in HRV were determined. The larger the area under the ROC curve, the greater the predictive power of HR for changes in HRV. Next, the cut-off points for HR, SBP, and DBP that obtained significant areas under the ROC curve were identified with their respective sensitivity and specificity values. The criterion for obtaining cut-off points for HR as a predictor of changes in HRV was considered as more balanced sensitivity and specificity.

In this study, HR, SBP, and DBP were considered good predictors when their CIs were greater than 0.50 and the area under the ROC curve was greater than 0.70. The CI determines whether the predictive ability of HR, SBP, and DBP is not due to chance and its lower limit must not be less than 0.50. A 95%CI was used in this study.

This cross-sectional study was conducted in agreement with the Declaration of Helsinki. The Human Research Ethics Committee of the Health Sciences Center, Federal University of Paraíba, João Pessoa (PB), Brazil, protocol n° 4.676.103, approved the study. All parents received information about the study and gave written informed consent before data collection.

## RESULTS

A total of 1,957 children were evaluated and classified according to BMI, with 1,009 males and 948 females. Of the total number of children, 79 (4.04%) were classified as underweight, 1,249 (63.82%) as normal weight, 328 (16.76%) as overweight, and 301 (15.38%) as obesity. This study included 125 of these children, of which 41 were normal weight and 81 were obese.

HR proved to be a good predictor of changes in the square root of the mean of the square of the differences between consecutive NN intervals (RMSSD) parameter in children of both sexes for the normal weight group (ROC 0.89; 95%CI 0.77–1.00) and obesity (ROC 0.90; 95CI% 0.83–0.97) (Table 1). HR was also a good predictor of changes in the RMSSD parameter in boys (ROC 0.97; 95%CI 0.88–1.00) and girls (ROC 0.84; 95%CI 0.62–1.00) from the normal-weight group, as well as in boys (ROC 0.91; 95%CI 0.80–1.00) and girls (ROC 0.90; 95%CI 0.79–1.00) from the obesity group (Table 1).

**Table 1:** The area under the receiver operating characteristic curve and 95% confidence interval, cut-off point, sensitivity, and specificity of heart rate as predictors of changes in parameters in heart rate variability indices in students from João Pessoa (PB), 2023.

Heart rate	ROC curve (95%CI)	Cut-off point (bpm)	Sensitivity (%)	Specificity (%)
Normal weight (girls+boys)				
RMSSD (ms)	0.89 (0.77–1.00)	93	100.00	77.78
LF/HF Ratio	0.88 (0.74–1.00)	94	90.91	87.50
SD2/SD1 Ratio	0.91 (0.81–1.00)	90	100.00	73.33
Normal weight (boys)				
RMSSD (ms)	0.97 (0.88–1.00)	93	100.00	87.50
LF/HF Ratio	1.00 (1.00–1.00)	102	100.00	100.00
SD2/SD1 Ratio	0.97 (0.89–1.00)	90	100.00	85.71
Normal weight (girls)				
RMSSD (ms)	0.84 (0.62–1.00)	94	100.00	70.00
LF/HF Ratio	0.77 (0.50–1.00)	94	85.71	75.00
SD2/SD1 Ratio	0.84 (0.62–1.00)	90	100.00	62.50
Obesity (girls+boys)				
RMSSD (ms)	0.90 (0.83–0.97)	93	84.62	78.26
LF/HF Ratio	0.77 (0.65–0.89)	94	75.68	68.00
SD2/SD1 Ratio	0.85 (0.75–0.94)	91	94.44	57.69
Obesity (boys)				
RMSSD (ms)	0.91 (0.80–1.00)	91	94.74	63.64
LF/HF Ratio	0.72 (0.52–0.92)	91	85.00	50.00
SD2/SD1 Ratio	0.87 (0.71–1.00)	91	95.24	77.78
Obesity (girls)				
RMSSD (ms)	0.90 (0.79–1.00)	94	85.00	83.33
LF/HF Ratio	0.81 (0.66–0.96)	94	82.35	66.67
SD2/SD1 Ratio	0.87 (0.74–0.99)	94	86.67	64.71

ROC: receiver operating characteristic; 95%CI: 95% confidence interval; area under the ROC curve that demonstrates discriminatory power for changes in heart rate variability parameters (lower limit of 95%CI > 0.50); RMSSD: square root of the mean of the square of the differences between consecutive NN intervals; LF/HF: low frequency/high frequency ratio; SD2/SD1: standard deviation of long-term continuous NN intervals/standard deviation of the instantaneous variability of continuous NN intervals in the Poincaré graph ratio.

The HR cut-off point for changes in the RMSSD parameter was 93 bpm for normal-weight boys (sensitivity 100.00% and specificity 87.50%) and 91 bpm for boys with obesity (sensitivity 94.74% and specificity 63.64%). In girls, the established cut-off was 94 bpm for both normal weight (sensitivity 100.00% and specificity 70.00%) and obesity (sensitivity 85.00% and specificity 83.33%).

Regarding changes in the low-frequency/high-frequency ratio (LF/HF) parameters, HR proved to be a good predictor for the normal weight students (ROC 0.88; 95%CI 0.74–1.00) and obesity group (ROC 0.77; 95%CI 0.65–0.89), regardless of gender. When considering gender, HR proved to be a good predictor for both boys (ROC 1.00; 95%CI 1.00–1.00) and girls (ROC 0.77; 95%CI 0.50–1.00) for the normal weight group, as for boys (ROC 0.72; 95%CI 0.52–0.92) and girls (ROC 0.81; 95%CI 0.66–0.96) of the obesity group (Table 1).

The HR cut-off points for changes in LF/HF were 102 bpm (100.00% sensitivity and 100.00% specificity) for normal-weight boys and 91 bpm (85.00% sensitivity and 50.00% specificity) for obese boys. In girls, the established cut-off point was 94 bpm for girls with normal weight (sensitivity 85.71% and specificity 75.00%) and obesity (sensitivity 82.35% and specificity 66.67%) (Table 1).

As for alterations in the parameter standard deviation of long-term continuous NN intervals /standard deviation of the instantaneous variability of continuous NN intervals in the Poincaré graph ratio (SD2/SD1), HR proved to be a good predictor in the group of normal-weight students (ROC 0.91; 95%CI 0.81–1.00) and in the group with obesity (ROC 0.85; 95%CI 0.75–0.94), regardless of gender. When considering gender, HR proved to be a good predictor for both boys (ROC 0.97; 95%CI 0.89–1.00) and girls (ROC 0.84; 95%CI 0.62–1.00) for the normal weight group, as for boys (ROC 0.87; 95%CI 0.71–1.00) and girls (ROC 0.87; 95%CI 0.74–0.99) of the obesity group (Table 1).

The HR cut-off points for changes in SD2/SD1 were 90 bpm (sensitivity 100.00% and specificity 85.71%) for boys with normal weight and 91 bpm (95.24% and specificity 77.78%) for boys with obesity. In girls, the established cut-off point was 90 bpm for girls with normal weight (sensitivity 100.00% and specificity 62.50%) and 94 bpm for obesity (sensitivity 86.67% and specificity 64.71%) (Table 1).

SBP proved to be a good predictor for changes in the RMSSD parameter in both sexes (ROC 0.81; 95%CI 0.64–0.97) and in boys (ROC 0.87; 95%CI 0.63–1.00), and for changes in the SD2/SD1 parameter in girls (ROC 0.77; 95%CI 0.51–1.00), in the group of normal-weight students. However, SBP did not prove to be a good predictor for the other parameters in the group of normal-weight students, as well as for any parameter in the group of students with obesity (Table 2). On the other hand, DBP proved to be a good predictor only for changes in the RMSSD parameter in normal-weight girls (ROC 0.81; 95%CI 0.56–1.00) (Table 3).

**Table 2:** The area under the receiver operating characteristic curve and 95% confidence interval, cut-off point, sensitivity, and specificity of systolic blood pressure as predictors of changes in parameters in heart rate variability indices in students from João Pessoa (PB), 2023.

Systolic blood pressure	ROC curve (95%CI)	Cut-off point (mmHg)	Sensitivity (%)	Specificity (%)
Normal weight (girls+boys)				
RMSSD (ms)	0.81 (0.64–0.97)	105	88.89	66.67
LF/HF Ratio	0.60 (0.37–0.83)	104	81.81	50.00
SD2/SD1 Ratio	0.64 (0.43–0.86)	106	75.00	66.67
Normal weight (boys)				
RMSSD (ms)	0.87 (0.63–1.00)	114	100.00	87.50
LF/HF Ratio	0.64 (0.21–1.00)	114	75.00	75.00
SD2/SD1 Ratio	0.53 (0.13–0.93)	114	60.00	71.43
Normal weight (girls)				
RMSSD (ms)	0.70 (0.42–0.97)	104	100.00	50.00
LF/HF Ratio	0.57 (0.25–0.89)	104	85.71	50.00
SD2/SD1 Ratio	0.77 (0.51–1.00)	106	85.71	75.00
Obesity (girls+boys)				
RMSSD (ms)	0.53 (0.37–0.68)	106	76.92	43.48
LF/HF Ratio	0.57 (0.41–0.72)	106	75.68	44.00
SD2/SD1 Ratio	0.59 (0.44–0.74)	105	80.56	46.15
Obesity (boys)				
RMSSD (ms)	0.51 (0.26–0.75)	106	84.91	45.45
LF/HF Ratio	0.50 (0.24–0.75)	106	85.00	40.00
SD2/SD1 Ratio	0. 59 (0.31–0.85)	106	85.71	44.44
Obesity (girls)				
RMSSD (ms)	0.54 (0.33–0.75)	106	65.00	50.00
LF/HF Ratio	0.60 (0.39–0.79)	106	64.71	53.33
SD2/SD1 Ratio	0.58 (0.37–0.78)	106	66.67	52.94

ROC: receiver operating characteristic; 95%CI: 95% confidence interval; area under the ROC curve that demonstrates discriminatory power for changes in heart rate variability parameters (lower limit of 95%CI >0.50); RMSSD: square root of the mean of the square of the differences between consecutive NN intervals; LF/HF: low frequency/high frequency ratio; SD2/SD1: standard deviation of long-term continuous NN intervals/standard deviation of the instantaneous variability of continuous NN intervals in the Poincaré graph ratio.

**Table 3:** The area under the receiver operating characteristic curve and 95% confidence interval, cut-off point, sensitivity and specificity of diastolic blood pressure as predictors of changes in parameters in heart rate variability indices in students from João Pessoa (PB), 2023.

Diastolic blood pressure	ROC curve (95%CI)	Cut-off point (mmHg)	Sensitivity (%)	Specificity (%)
Normal weight (girls+boys)				
RMSSD (ms)	0.61 (0.39–0.83)	62	88.89	61.11
LF/HF Ratio	0.50 (0.27–0.72)	62	63.64	50.00
SD2/SD1 Ratio	0.55 (0.32–0.79)	62	66.67	53.33
Normal weight (boys)				
RMSSD (ms)	0.34 (0.02–0.67)	59	100.00	12.50
LF/HF Ratio	0.25 (0.00–0.54)	59	100.00	12.50
SD2/SD1 Ratio	0.30 (0.00–0.62)	59	100.00	14.29
Normal weight (girls)				
RMSSD (ms)	0.81 (0.56–1.00)	62	100.00	80.00
LF/HF Ratio	0.71 (0.40–1.00)	58	85.71	50.00
SD2/SD1 Ratio	0.75 (0.47–1.00)	61	85.71	62.50
Obesity (girls+boys)				
RMSSD (ms)	0.52 (0.37–0.66)	56	82.05	17.39
LF/HF Ratio	0.56 (0.41–0.70)	56	81.08	16.00
SD2/SD1 Ratio	0.60 (0.45–0.74)	56	86.11	23.08
Obesity (boys)				
RMSSD (ms)	0.51 (0.28–0.74)	57	84.21	18.18
LF/HF Ratio	0.57 (0.35–0.79)	56	85.00	10.00
SD2/SD1 Ratio	0.58 (0.36–0.81)	56	90.48	22.22
Obesity (girls)				
RMSSD (ms)	0.51 (0.30–0.72)	59	75.00	25.00
LF/HF Ratio	0.54 (0.34–0.75)	59	76.47	20.00
SD2/SD1 Ratio	0.60 (0.39–0.80)	59	80.00	29.41

ROC: receiver operating characteristic; 95%CI: confidence interval; area under the ROC curve that demonstrates discriminatory power for changes in heart rate variability parameters (lower limit of 95%CI >0.50); RMSSD: square root of the mean of the square of the differences between consecutive NN intervals; LF/HF: low frequency/high frequency ratio; SD2/SD1: standard deviation of long-term continuous NN intervals/standard deviation of the instantaneous variability of continuous NN intervals in the Poincaré graph ratio.

The SBP cut-off points for screening changes in the RMSSD parameter for both sexes was 105 mmHg (sensitivity 88.89% and specificity 66.67%) and for normal-weight boys, it was 114 mmHg (sensitivity 100% and specificity 87.50%). For the SD2/SD1 parameter in normal-weight girls, the SBP cut-off point as a screening tool was 106 mmHg (sensitivity 85.71% and specificity 75.00%). The DBP cut-off point for screening changes in the RMSSD parameter in normal-weight girls was 62 mmHg (sensitivity 100.00% and specificity 80.00%).

**Figure 1:**
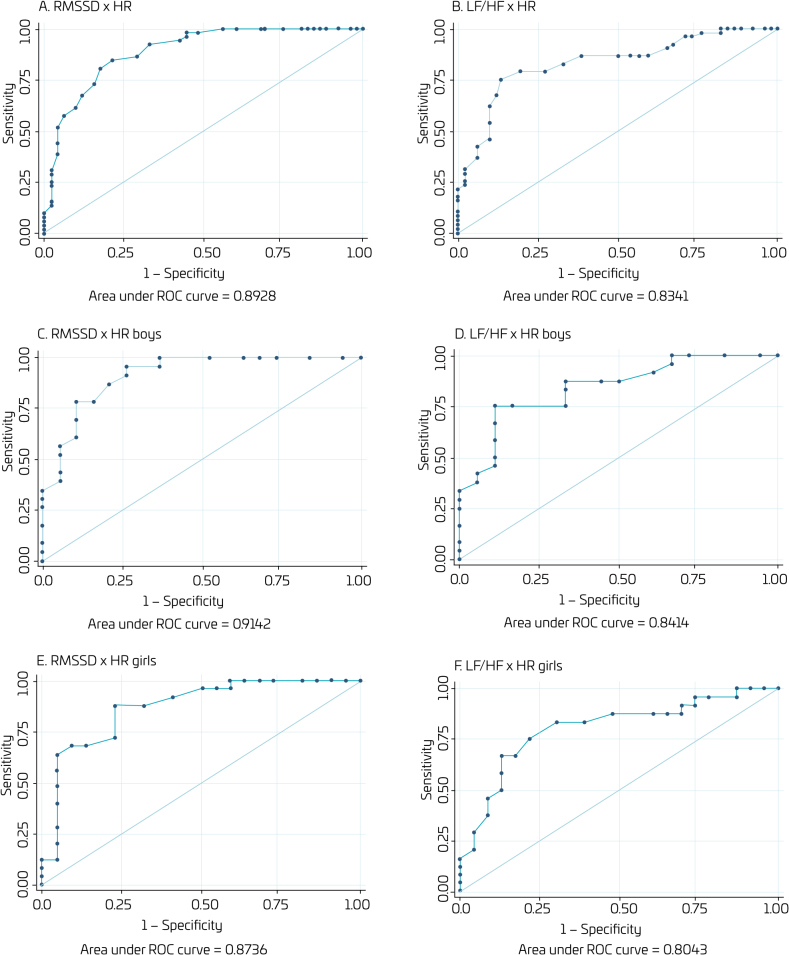
Receiver operating characteristic (ROC) curve for general and by sex analyses of the heart rate with RMSSD and LF/HF in children from João Pessoa (PB), 2023.

ROC: receiver operating characteristic; HR: heart rate; RMSSD: square root of the mean of the square of the differences between consecutive NN intervals; LF/HF: low frequency/high frequency ratio; SD2/SD1: standard deviation of long-term continuous NN intervals/standard deviation of the instantaneous variability of continuous NN intervals in the Poincaré graph ratio.

The results of general and by sex analysis of area under the ROC curve of HR to RMSSD and LF/HF indices in children are available in Figure 1.

## DISCUSSION

The results of this study suggest that HR may be a feasible measure for predicting HRV changes and that established cut-off points may be useful in the clinical practice of pediatricians and health care professionals to identify children at risk for cardiac autonomic dysfunction. In addition, the findings showed that SBP and DBP can also be used as important screening parameters, but there are differences according to the stratifications. The use of HRV as a tool for estimating cardiovascular risk on a large scale has become unfeasible so far due to the need for more sophisticated equipment that is not yet available in basic public health units in Brazil. On the other hand, the use of HR as a cardiovascular risk marker and predictor of metabolic dysfunction in children^22^ may be interesting given its relative practicality of measurement.

The established cut-off point determines the sensitivity and specificity of the analyzed tool, i.e., if the cut-off point is low, many patients with low cardiovascular risk could be identified as having cardiovascular risk, leading to low sensitivity and specificity. On the other hand, if the cut-off point is high, some of the patients with cardiovascular risk may be diagnosed as having low risk, characterizing the test as having low sensitivity and high specificity. Therefore, it is essential to select the cut-off point with high sensitivity and specificity to reduce the risk of false-positive and false-negative diagnoses.^23,24^

It is recognized that resting HR is associated with BP and metabolic dysfunctions in children and adolescents.^22, 25^ To our better understanding, this is the first report demonstrating that HR and BP can be used to predict HRV change in children. Considering that HRV is a prediction of autonomic dysfunction^26^ and cardiovascular events,^27^ the findings of the present study suggest that normal ranges of HR established for children in systematic reviews of observational studies^28^ should be interpreted with caution in clinical practice with regard to its effect on HRV.

Early studies evaluated other variables as predictors of cardiovascular risk in children and established cut-off points. Hand grip strength normalized for body mass was evaluated as a cut-off point for cardiovascular risk in Chilean children. In this case, the authors established 0.33 and 0.40 as cut-off points for boys and girls, respectively.^29^ Another study evaluated the serum concentrations of vitamin D and determined the cut-off points for cardiovascular risk in children. According to the authors, vitamin D had a good predictive capacity for cardiovascular risk, with the cut-off point for predicting this risk being 32 ng/ml.^30^ The use of these different variables evaluated in clinical practice can provide a more robust diagnosis of cardiovascular risk for children.

The cross-sectional design does not provide support for causality statements. Therefore, prospective studies on children are needed to better characterize the relationship between HR and HRV, and the convenience sample is a limitation of the present study. In summary, we conclude that the predictive validity of HR was shown to be at a good level with high sensitivity and acceptable specificity for the cut-off points according to the different analyses stratified by gender and nutritional status. Health professionals will be able to use the HR to estimate cardiovascular risk in children of different sexes and nutritional status. Furthermore, it is necessary to test the predictive validity of HR for cardiovascular risk in another population.

## Data Availability

The database that originated the article is available with the corresponding author.
